# The Interrater and Intrarater Reliability of the Humeral Head Ossification System and the Proximal Femur Maturity Index Assessments for Patients with Adolescent Idiopathic Scoliosis

**DOI:** 10.3389/fped.2023.1131618

**Published:** 2023-03-09

**Authors:** Huan Wang, Qing-da Lu, Chen-xin Liu, Shuai Yang, Bo-hai Qi, Huan-an Bai, Ji-ning Qu, Ye Yang, Xiao-hui Jin, Ming Yang, Fei Su, Ya-ting Yang, Qiang Jie

**Affiliations:** ^1^ Pediatric Orthopedic Hospital, Honghui Hospital, Xi’an Jiaotong University, Xi'an, China; ^2^ Department of Pediatric Surgery, Baoji Maternal and Child Health Care Hospital, Baoji, China; ^3^ Department of Radiology, Honghui Hospital, Xi'an Jiaotong University, Xi'an, China

**Keywords:** Humeral Head Ossification System, Proximal Femur Maturity Index, adolescent idiopathic scoliosis, reliability, skeletal maturity

## Abstract

**Background:**

Skeletal maturity can evaluate the growth and development potential of children and provide a guide for the management of adolescent idiopathic scoliosis (AIS). Recent studies have demonstrated the advantages of the Humeral Head Ossification System (HHOS) and the Proximal Femur Maturity Index (PFMI), based on standard scoliosis films, in the management of AIS patients. We further assessed the HHOS and the PFMI method's reliability in the interrater and intrarater.

**Methods:**

The data from 38 patients, including the humeral head and proximal femur on standard scoliosis films, were distributed to the eight raters in the form of a PowerPoint presentation. On 38 independent standard spine radiographs, raters utilized the HHOS and PFMI to assign grades. The PPT sequence was randomly changed and then reevaluated 2 weeks later. For every system, the 95% confidence interval (95% CI) and intraclass correlation coefficient (ICC) were calculated to evaluate the interrater and intrarater reliability.

**Results:**

The HHOS was extremely reliable, with an intraobserver ICC of 0.802. In the first round, the interobserver ICC reliability for the HHOS was 0.955 (0.929–0.974), while in the second round, it was 0.939 (0.905–0.964). The PFMI was extremely reliable, with an intraobserver ICC of 0.888. In the first round, the interobserver ICC reliability for the PFMI was 0.967 (0.948–0.981), while in the second round, it was 0.973 (0.957–0.984).

**Conclusions:**

The HHOS and PFMI classiﬁcations had excellent reliability. These two methods are beneficial to reduce additional exposure to radiation and expense for AIS. There are advantages and disadvantages to each classification. Clinicians should choose a personalized and reasonable method to assess skeletal maturity, which will assist in the management of adolescent scoliosis patients.

## Introduction

Skeletal maturity can evaluate the growth and development potential of children and provide a guide for the treatment of adolescent idiopathic scoliosis (AIS). A convenient and reliable method for assessing skeletal maturity is of great significance for guiding the treatment of spinal curvatures, especially in idiopathic scoliosis (1). By using skeletal maturity as a predictor, clinicians may determine the likelihood that a scoliosis curve will progress, which helps them decide how long to have their patients wear braces and whether to operate.

Currently, there are several commonly used methods to assess bone maturity: Greulich and Pyle (2, 3), Tanner–Whitehouse III (TW3) (4, 5), Sanders system (6, 7), Thumb Ossification Composite Index (TOCI) (8, 9), Humeral Head Ossification System (HHOS) (10, 11), Proximal Femur Maturity Index (PFMI) (12), and Risser's sign (13). Although the Risser sign can be used as an indicator of skeletal maturity evaluation, it has suboptimal reliability. These methods, for example, the Greulich and Pyle, Tanner–Whitehouse III, Thumb Ossification Composite Index, and the more acceptable Sanders system, evaluate skeletal maturity by hand radiographs. The Sanders system has substituted the Risser system to some extent. The TW3 score and Greulich and Pyle systems are clinically complicated, as they require access to atlas for individual bone scores. The TW3 score and Greulich and Pyle can be used reliably in idiopathic scoliosis patients with artificial intelligence (AI) model assistance (3, 5). Many studies (14, 15) indicate that the Sanders system and TOCI can be used as relatively simple and reliable methods in patients with idiopathic scoliosis. These methods of assessing bone maturity require x-rays of the hand. Many studies (16, 17) have shown a correlation between radiation exposure and cancer risk. However, scoliosis patients require long-term follow-up, usually a full-length spine film every 6 months. The adoption of a skeletal maturity assessment system without additional radiation is what is expected (18). HHOS and PFMI are used to assess bone growth and development by measuring the humerus head and proximal femur epiphysis included in standard spine radiographs.

The Humeral Head Ossification System was first proposed by Li et al. (11) to classify the proximal humerus in the radiographs of 94 scoliosis patients. In addition, developers have confirmed the extreme reliability of the HHOS (11). However, the system needs further confirmation of its reliability at other institutions not participating in the research and development. Lopyan et al. (18) repeated experiments based on the study by Li et al. to find fair to moderate interrater and intrarater reliability. This distinction requires further investigation. Cheung et al. (12) first described the Proximal Femur Maturity Index, and reliability testing of the PFMI revealed excellent intraobserver and interobserver agreement. Similarly, the Proximal Femur Maturity Index can be reproducible. This study assesses HHOS’s and PFMI’s reliability in the interrater and intrarater.

## Materials and methods

**Figure 1:**
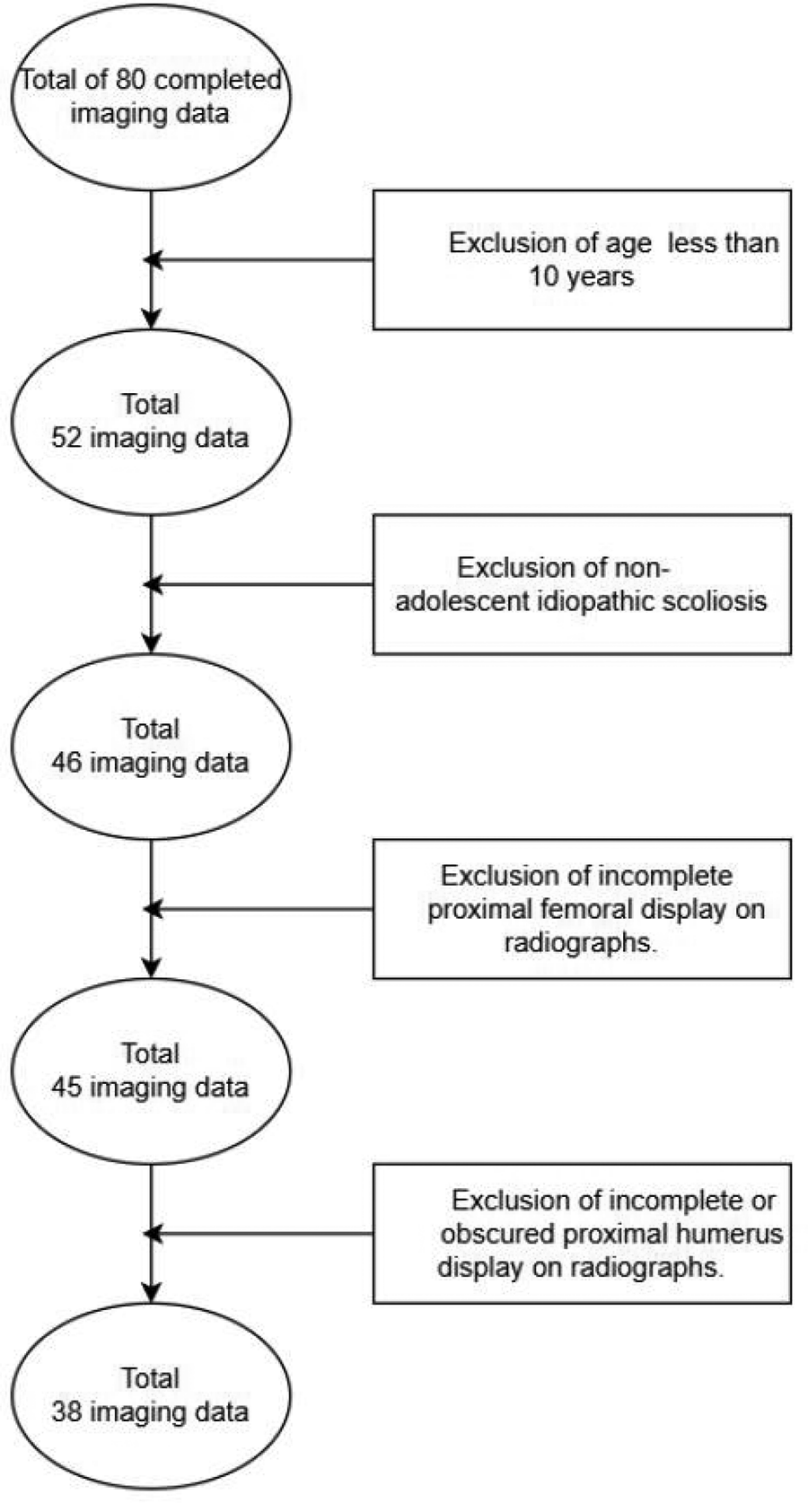
Inclusion and exclusion criteria flow chart.

This study was a retrospective analysis of 80 patients from the Xi’an Honghui Hospital. After obtaining approval from the institutional ethics committee, we obtained the data of 38 patients with AIS by inclusion and exclusion criteria (Figure 1). These data included demographic information and x-rays of patients who met the inclusion and exclusion criteria. Study inclusion criteria: patients diagnosed with AIS, between the ages of 10 and 18, and with available posteroanterior (PA) scoliosis films that included the humeral head and the proximal femur. Exclusion criteria: non-adolescent idiopathic scoliosis (for example, early-onset scoliosis) and patients with incomplete films (excluding the humeral head and proximal femur). Demographic data consist of gender and age. Each rater received a PowerPoint (PPT) presentation consisting of radiographs. The PPT sequence was randomly changed and then reevaluated 2 weeks later. The raters were eight independent observers from the Xi’an Honghui Hospital and the Baoji Maternal and Child Healthcare Hospital. The seven raters from the Xi’an Honghui Hospital comprised one chief pediatric orthopedic surgeon, one deputy chief pediatric orthopedic surgeon, two attending pediatric orthopedic surgeons, two pediatric orthopedic surgeons, and one radiologist. The other rater was an attending pediatric orthopedic surgeon from another hospital. There are four stages of physician titles in the Chinese healthcare system: primary title (Resident Physician), middle title (Attending Physician), vice-senior title (Deputy Chief Physician), and senior title (Chief Physician). In general, each level of physician needs a 5-year interval of clinical accumulation to pass the qualification and examination before being promoted to the next level. The x-rays are required to be independently categorized by the raters. After a brief study, all raters who had not previously learned the two classification systems evaluated skeletal maturity using the HHOS and PFMI systems.

**Figure 2:**
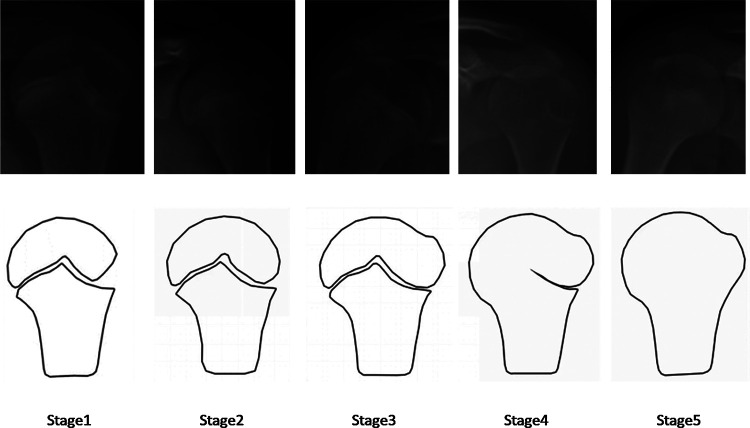
Brief characterization of the HHOS grades. Stage 1: The metaphyseal is wider than the epiphysis, and there is a space at the epiphysis that resembles a triangle; Stage 2: The lateral margin of the epiphysis becomes progressively more smooth; Stage 3: The epiphysis and metaphysis are basically in line; Stage 4: gradually fusion of physis from medial to lateral; Stage 5: the humeral head epiphyseal and metaphyseal are completely fused (11). HHOS, Humeral Head Ossification System.

**Figure 3:**
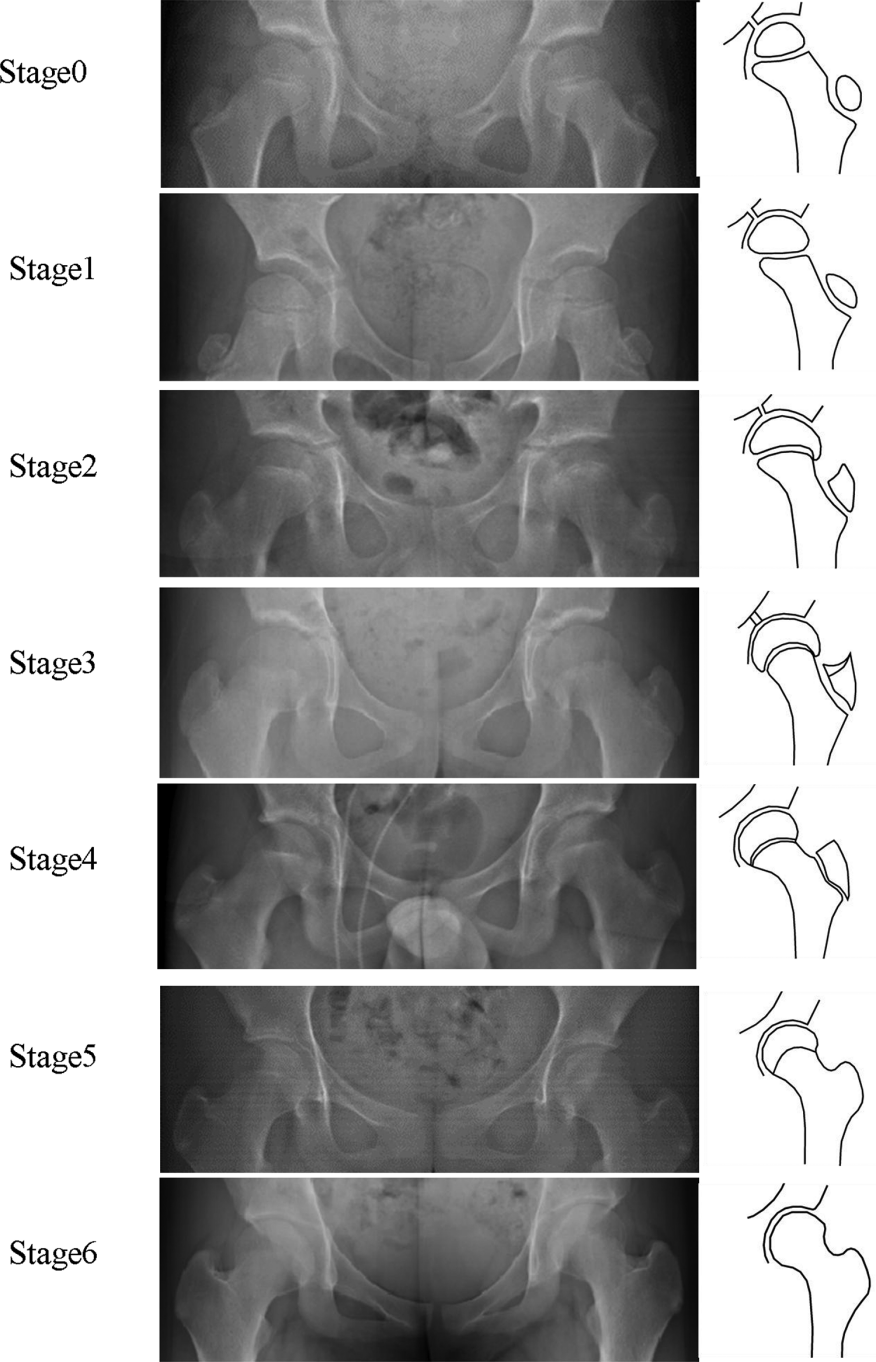
Brief characterization of the PFMI grades. Stage 0: The metaphysis is wider than the femoral head epiphysis, with a smooth epiphysis at the greater trochanter; Stage1: The metaphysis is narrower than the femoral head epiphysis, with a tapered shape at the greater trochanter; Stage 2: The lateral edge of the femoral epiphysis is beaked, with a triangular shape at the greater trochanter; Stage 3: The medial edge of the femoral epiphysis is beaked, with a double contour line at the greater trochanter; Stage 4: The femoral head physis narrows and the epiphysis at the greater trochanter begins to fuse in the middle; Stage 5: Almost fusion of the femoral head epiphysis and complete fusion of the epiphysis at the greater trochanter; Stage 6: Complete fusion of the epiphysis at the femoral head and greater trochanter. The triangular cartilage is open in Stages 0–2, open or closed in Stage 3, and closed in Stages 4–6 (12). PFMI, Proximal Femur Maturity Index.

The HHOS classification (11) assesses skeleton maturity by epiphyseal morphology of the humeral head, using the developer's model as guidance. The HHOS classification is classified into five stages (1–5) (Figure 2). PFMI classification (12) assesses skeletal maturity by the comprehensive growth and development of the femoral head morphology, greater trochanter, and triangular cartilage, using the developer's model as guidance. The PFMI classification is classified into seven stages (0–6) (Figure 3).

We analyzed the data using IBM SPSS 26 statistical software. The intraclass correlation coefficient (ICC) was calculated using a random effects model with an absolute agreement to identify the intraobserver and interobserver reliability for each rater. The intraclass correlation coefficient of less than 0.20 denotes poor agreement, one between 0.20 and 0.40 denotes fair agreement, one between 0.40 and 0.60 denotes moderate agreement, one between 0.60 and 0.80 denotes good agreement, and one of more than 0.80 denotes excellent agreement. ICC was also counted for each grader to estimate intraobserver reliability.

## Results

The demographic data of 38 patients were analyzed statistically in this study (mean age, 12.55 ± 1.927 years; 76.3% female).

### HHOS interreliability and intrareliability

The results of the intrarater and interrater reliability of the HHOS system are described in Table 1. The HHOS had excellent reliability for intrarater and interrater agreements. The intrarater reliability analysis for each grader is described in Table 2. All rater intrarater agreements were excellent. Each grader's intrarater agreement varied from moderate to excellent.

**Table 1:** Reliability results for the HHOS and PFMI.

Type	Intrarater reliability	Trial	Interrater reliability	95% CI
HHOS	0.802	1	0.955	0.929–0.974
		2	0.939	0.905–0.964
PFMI	0.888	1	0.967	0.948–0.981
		2	0.973	0.957–0.984

95% CI, 95% conﬁdence interval; HHOS, Humeral Head Ossification System; PFMI, Proximal Femur Maturity Index.

**Table 2:** Each intrarater reliability results for HHOS and PFMI.

Grader^a^	HHOS		PFMI	
	Reliability	95% CI	Reliability	95% CI
Chief physician^b^	0.898	0.813–0.946	0.939	0.886–0.968
Deputy chief physician^b^	0.682	0.467–0.821	0.852	0.734–0.920
Attending physician^b^	0.932	0.873–0.964	0.893	0.803–0.943
Attending physician^b^	0.582	0.326–0.759	0.755	0.577–0.865
Attending physician^c^	0.896	0.808–0.944	0.832	0.701–0.909
Radiologist^b^	0.972	0.947–0.985	0.982	0.966–0.991
Resident physician^b^	0.685	0.471–0.823	0.971	0.946–0.985
Resident physician^b^	0.768	0.596–0.872	0.876	0.774–0.933

95% CI, 95% confidence interval; HHOS, humeral head ossification system; PFMI, proximal femur maturity index.

^a^ The grader has listed a ranking in order of highest to lowest position; There are four stages of physician titles in the Chinese healthcare system: primary title (Resident Physician), middle title (Attending Physician), vice-senior title (Deputy Chief Physician), and senior title (Chief Physician). In general, each level of physician needs a 5-year interval of clinical accumulation to pass the qualification and examination before being promoted to the next level.

^b^ The rater is from the Honghui Hospital.

^c^ The rater is from the Baoji Maternal and Child Healthcare Hospital.

### PFMI interreliability and intrareliability

The results of the intrarater and interrater reliability of the PFMI system are described in Table 1. The PFMI had excellent reliability for intrarater and interrater agreements. The intrarater reliability analysis for each grader is described in Table 2. Compared to HHOS, each grader's intrarater agreement varied from good to excellent, and PFMI seemed to have a more stable agreement than HHOS.

## Discussion

Assessing the skeleton maturity for AIS plays an important role in knowing the advancement of the disease and intervening effectively (14). The risk of AIS progression is substantially correlated with bone growth potential. The peak height velocity can be used to determine the remaining potential of bone growth to determine the most appropriate treatment. Skeletal maturity reliability assessment is still a significant procedure. Given multiple existing methods of assessing bone growth potential, this study provides data support for HHOS and PFMI systems.

While many methods have been proposed to evaluate skeletal maturity, all of these methods currently in use have disadvantages and advantages (18). Overall, Risser's sign has low interobserver and intraobserver reliability. The Greulich–Pyle and Tanner–Whitehouse classifications are more adapted for academic studies and have some difficulties in clinical work (14). The TOCI and Sanders systems, which are relatively simple to operate, have a fast learning curve and high reproducibility and reliability, but they need additional hand films. The HHOS and PFMI systems have multiple stages that also correlate with growth potential, and standard PA scoliosis radiographs usually obtain information about HHOS and PFMI (18).

The developer's study described interrater and intrarater reliability as excellent for HHOS among orthopedic surgeons with different experience (10, 11). The system used intraclass correlation coefficients to evaluate intraobserver and interobserver reliability. The developer's research suggested that the humerus’ location is not constrained (11). However, we discovered that numerous humeral heads, which had imaging overlays or occlusions on the humeral head, could not be used to assess skeletal maturity on standard scoliosis films. We concentrated on including optimal graphics to evaluate the reliability of the HHOS. The impact of different humeral head positions on the assessment of the HHOS system should be the subject of future study. It is crucial that evaluation systems be tested for reliability and repeatability by other institutions that do not take part in its development. Lopyan et al (18) repeated experiments based on the original study to find fair to moderate interrater and intrarater reliability. In light of the original study, we replicated the experiment and discovered good agreement in terms of both interobserver and intraobserver reliability for HHOS. This suggests that other institutions are required to confirm the value of this classification.

Cheung et al. originally described the Proximal Femur Maturity Index (12). The PFMI classification (12) assesses skeletal maturity by the comprehensive growth and development of the femoral head morphology, greater trochanter, and triangular cartilage. Furthermore, the PFMI showed fair to good agreement between interobserver, with excellent intraobserver reliability. The developers provided a flowchart and made the appropriate modifications to their classification. After 3 months, the rater reevaluated it and discovered excellent intraobserver and interobserver reliability. The kappa coefficient was employed to assess intraobserver and interobserver reliability for PFMI. This study expands the body of work of Cheung et al. proving excellent interrater and intrarater reliability. The PFMI classification appears to be more readily available on standard scoliosis radiographs. However, it is worth thinking about the effects of radiation on the gonads. In conclusion, we prefer to utilize the straightforward approach in clinical work (18). There are advantages and disadvantages to each classification. We should choose a personalized and reasonable strategy based on the situation of adolescent idiopathic scoliosis. There is currently a small amount of literature indicating the reliability of this classification scheme, which needs to be evaluated through additional studies.

## Limitations

We enumerate several limitations. First, this was a retrospective cohort study to assess the reliability of different classifications by collecting historical radiographs. This study was limited to adolescent idiopathic scoliosis, and further studies are needed for scoliosis in other age groups. Second, this study included only x-rays with the best images and did not include humeral head morphology in all positions because some of the humeral head was overlapping or obscured on the films. Future studies should assess the reliability of the HHOS in different humeral head morphologies. Finally, the relationship between HHOS and PFMI should be the subject of future study.

## Conclusions

The HHOS and PFMI classifications had excellent reliability. These two methods are beneficial to reduce additional exposure to radiation and expense for AIS. There are advantages and disadvantages to each classification. Clinicians should choose a personalized and reasonable method to assess skeletal maturity, which will assist in the management of adolescent scoliosis patients.

## Data Availability

The original contributions presented in the study are included in the article/Supplementary Material, further inquiries can be directed to the corresponding author.
